# Attention Performance Correlated With White Matter Structural Brain Networks in Shift Work Disorder

**DOI:** 10.3389/fpsyt.2021.802830

**Published:** 2022-02-01

**Authors:** Yanzhe Ning, Meng Fang, Yong Zhang, Sitong Feng, Zhengtian Feng, Xinzi Liu, Kuangshi Li, Hongxiao Jia

**Affiliations:** ^1^The National Clinical Research Center for Mental Disorders & Beijing Key Laboratory of Mental Disorders, Beijing Anding Hospital, Capital Medical University, Beijing, China; ^2^Advanced Innovation Center for Human Brain Protection, Capital Medical University, Beijing, China; ^3^Dongzhimen Hospital, Beijing University of Chinese Medicine, Beijing, China

**Keywords:** attention, shift work disorder, structural brain network, graph analysis, diffusion tensor imaging (DTI)

## Abstract

Neuroimaging studies have revealed that shift work disorder (SWD) affected the functional connectivity in specific brain regions and networks. However, topological disruptions in the structural connectivity of the white matter (WM) networks associated with attention function remain poorly understood. In the current study, we recruited 33 patients with SWD and 29 matched healthy subjects. The attention network test (ANT) was employed to investigate the efficiency of alerting, orienting, and executive control networks. The diffusion tensor imaging (DTI) tractography was used to construct the WM structural networks. The graph theory analysis was applied to detect the alterations of topological properties of structural networks. Our results showed lower alerting effect and higher executive effect for patients with SWD. Using the link-based analysis, 15 altered connectivity matrices (lower fiber numbers) were found between the two groups. Meanwhile, the graph theoretical analysis showed that the global efficiency and characteristic path length within SWD patients declined in contrast with the healthy controls. Furthermore, a significantly negative correlation was found between the executive effect and global network efficiency. Our findings provide the new insights into the fundamental architecture of interregional structural connectivity underlying attention deficits in SWD, which may be a potential biomarker for SWD.

## Introduction

Shift work disorder (SWD), involving circadian rhythm disorders, is characterized by the difficulty in falling asleep, sufficient sleep, and daytime fatigue, which result in the altered cognitive performance (1). One study has shown that the exposure duration of shift work was negatively associated with impaired cognition (2). Another study on the effects of shift work nurses on sleep and cognitive function has shown impaired attention for SWD (3). However, the knowledge about underlying mechanisms of the cognitive impairments on SWD is limited.

Attention network test (ANT), developed by Fan et al. (4), provides an easy way to distinguish three independent attentional components within one integrated task. It is widely used to measure the attentional performance of healthy individuals and mental patients (5–7). The ANT study on sleep deprivation has shown an overall slowing of reaction times in the nocturnal session, along with impairments in orientation and executive function (8). However, the abnormal attention on SWD *via* the ANT is still unknown.

To date, numerous studies on functional abnormalities in SWD have been detected *via* functional magnetic resonance imaging (fMRI) (9, 10). One study on night shift nurses has shown higher ReHo in the bilateral occipital lobe and left parietal lobe and lower regional homogeneity (ReHo) in the bilateral cerebellar hemisphere in contrast with the day shift nurses (10). Another study on SWD has revealed disrupted functional connectivity between default mode network and sensorimotor network, left frontoparietal network, and salience network (9). Nevertheless, there is no study focusing on structural changes in SWD. The diffusion tensor imaging (DTI) opens a window to investigate the brain structural connectivity *in vivo* (11). In combination with a graph theoretical method, this advanced technique can offer insight into the brain's structural connection patterns. In recent years, graph theory has been widely used to analyze large-scale brain networks across the whole brain. Previous neuroimaging studies have brought the analysis of structural large-scale brain networks to healthy subjects (12), primary insomnia (13), and so on. One study on patients with depression has revealed the decreased shortest path length and clustering coefficient and increased global and local efficiency (14). Another study on primary insomnia has suggested that the insomnia patients showed increased local efficiency and decreased global efficiency (13). However, to our knowledge, the structural large-scale brain networks on SWD remain unexplored.

In the current study, we applied DTI tractography in combination with graph theory to examine the neuroanatomical substrates of the three attention systems measured by ANT in 33 patients with SWD and 29 healthy subjects. First, white matter connectivity, assessed with diffusion tensor imaging deterministic tractography, was modeled as a structural network comprising 148 nodes defined by the Destrieux atlas. Then, we calculated global, local, and regional efficiencies of structural brain networks for each subject. Finally, we conducted the linear regression analyses to investigate the relationship between the network efficiency and the three attentional effects.

## Materials and Methods

The Beijing Anding Hospital of Ethics Committee approved this study. All participants signed informed consents prior to participation.

### Participants

Thirty-three right-handed participants (two male, aged 28.06 ± 2.28 years) were diagnosed with SWD in accordance with the International Classification of Sleep Disorders (2nd Edition) by the American Sleep Disorders Association; nursing staff at Beijing Anding Hospital; 20–40 years old, right-handed; working regular night shift for at least 1 year and at least two shifts per week; with no history of prophylactic or therapeutic medicine in the past 3 months; and no history of long-term analgesic use. The exclusion criteria were as follows: being pregnant or breastfeeding, having a history of neurological or psychiatric disorders, participating in cognitive experiments within 1 year, any mental or physical impairments that may interfere with participation, any brain structure damage or abnormalities identified by MRI examinations, any history of alcohol or drug dependence, and any MRI contraindications.

Another 29 healthy subjects (three male, aged 27.17 ± 2.25 years) were recruited meeting the following inclusion criteria: relative regularity of sleep in the past 12 months; aged 20–40 years, right-handed; sleeping <3 times per month after 2,300 h, and working the night shift <3 times per month in the past year.

### ANT Assessment

ANT, a cognitive task designed by Fan et al. (4) investigated the efficiency of alerting, orienting, and executive control networks involved in attention. All recruited participants were ordered to press a button as accurately and rapidly as possible to determine the direction of the target. Participants were presented with the target and flankers until they made a response or 2,000 ms had elapsed. A cue would be presented for 200 ms prior to the target. The task used three cue conditions: no cue, center cue, and spatial cue. Each participant completed three blocks in this experiment, each block lasting for 5 min and 42 s and consisting of 36 trials, plus two buffer trials at the start. In each block, a total of six trial types were presented in a counterbalanced order. All subjects were trained before the formal experiment. Stimuli were presented, and behavioral responses were recorded using E-Prime 2.0 software. Three attention networks were evaluated by calculating ratio scores of alerting, orienting, and EC issues. The formulas were as follows:

Alerting effect = no cue response – center cue response.
Orienting effect = center cue response – spatial cue response.
Executive effect = incongruent target response – congruent target response.

**Figure 1:**
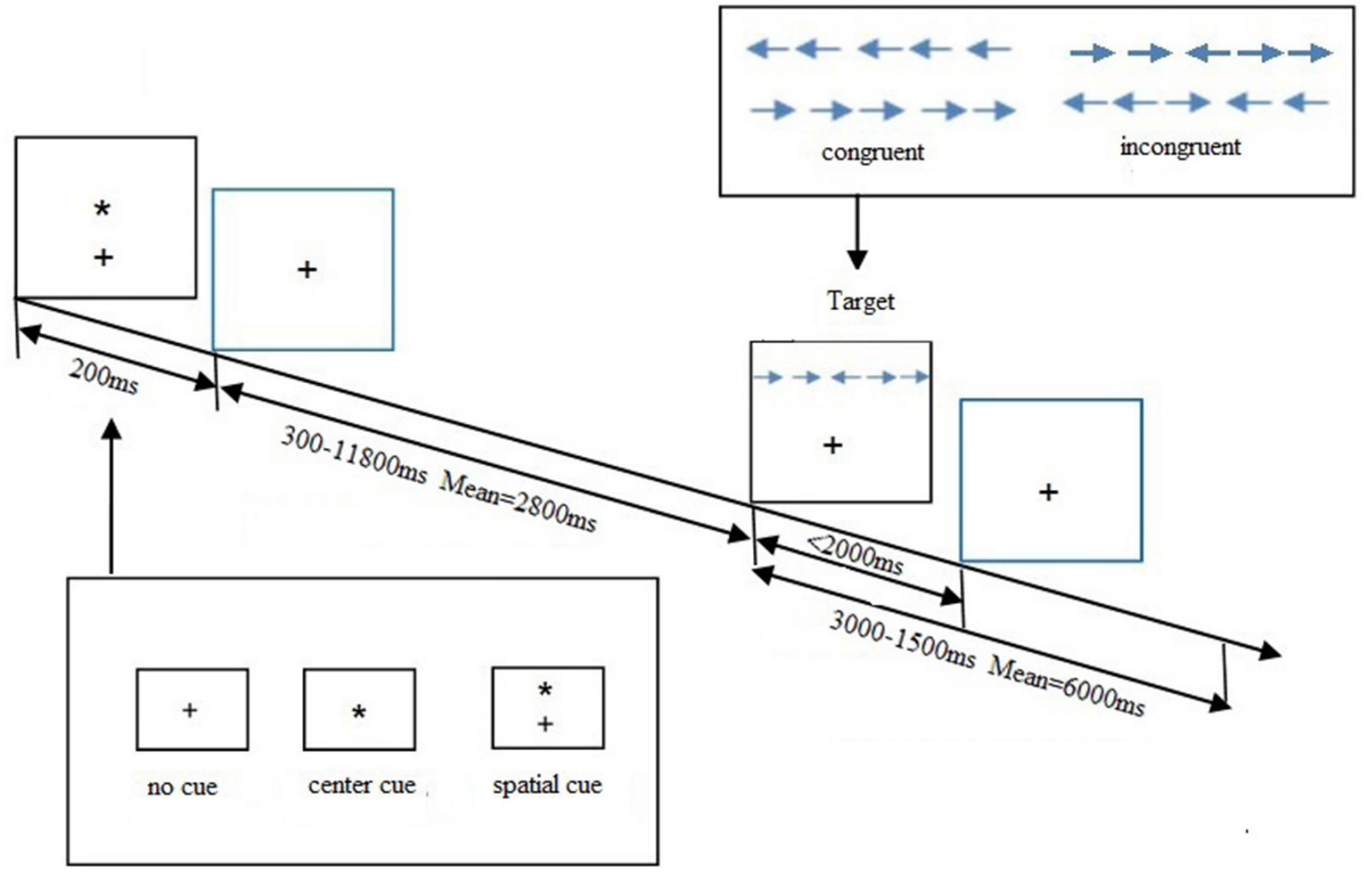
Attention network test (ANT) schematic showing the time and different conditions for each trial. A trial starts *via* presenting one cue condition for 200 ms. A variable delay period of 300–11,800 ms elapses before one target condition is displayed. The target disappears after 2,000 ms had elapsed or until they made a response. From the moment, the target appears the trial continues for a variable delay period of 3,000–15,000 ms.

A higher executive effect score reflected a relatively poorer executive function. The total accuracy of each subject was calculated, and those with over 20% error rates should be excluded from this study. The trials with incorrect responses or with response time (RT) longer than 1,500 ms or shorter than 200 ms were also excluded to avoid the influence of the outliers. The procedure of ANT is shown in Figure 1.

### MRI Acquisition

The MRI scan was acquired using a 3.0-T MRI scanner (Siemens, Prisma Germany) at Anding Hospital, Beijing, China. Participants were instructed to rest for 30 min before scanning, stay still, stay focused, keep their eyes closed, and refrain from falling asleep during the scan. Earplugs were worn to reduce scanner noise. The foam head holders were immobilized to minimize head movements during scanning.

Prior to the DTI scanning, a standard 3D T1-weight high-resolution structural image was acquired with the following parameters: voxel size = 1 mm^3^, TR = 2,530 ms, TE = 3.39 ms, flip angle = 90°, matrix = 256 × 256, field of view =256 mm × 256 mm, slice thickness = 1 mm. The DTI data lasted 12 min and 30 s with a single-shot, echo-planar imaging sequence. The diffusion sensitizing gradients were applied along 64 non-collinear directions (*b* = 1,000 s/mm^2^) with an acquisition without diffusion weighting (*b* = 0 s/mm^2^). In addition, specific parameters were as follow: TR= 11,000 ms, TE = 98 ms, matrix = 128 × 128, field of view = 256 mm × 256 mm, slice thickness = 2.0 mm with no gap.

### T1 Data Preprocessing

All T1 data were preprocessed by the Freesurfer 6.0 (http://surfer.nmr.mgh.harvard.edu) with the basic method of recon-all to remove the nonbrain structure. Moreover, 5ttgen from MRtrix was applied to generate five-tissue-type image, including cortical gray matter, subcortical gray matter, white matter, cerebrospinal fluid, and pathological tissues. Then, the parcellation of cortical ribbon was segmented into 148 different regions by the Destrieux atlas (15, 16). After that, MRtrix was used to convert the parcellated image for producing 164 nodes, which was prepared for connectome analysis.

### DTI Data Preprocessing and Tractography

**Figure 2:**
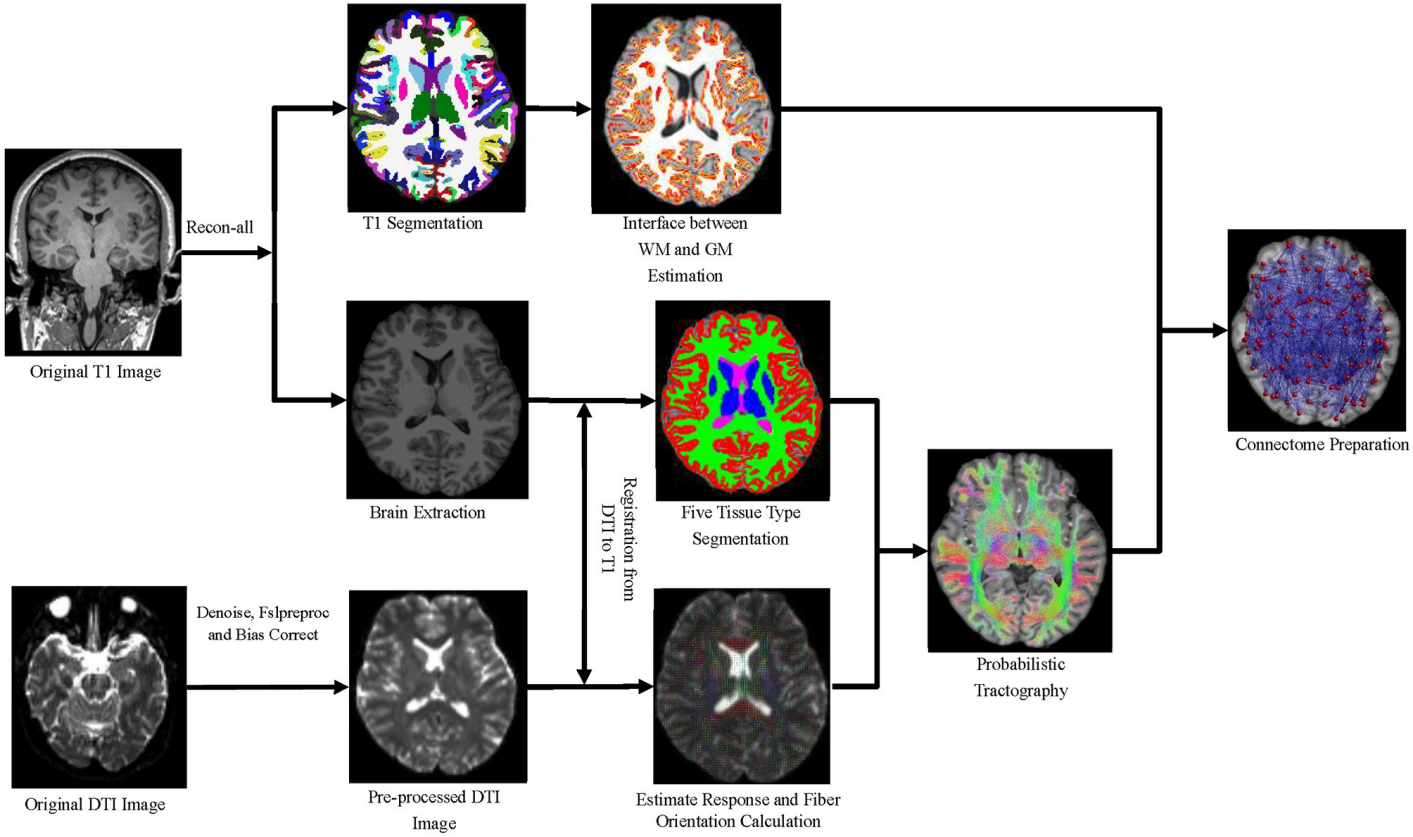
A flowchart of white matter network construction.

First, the noise was removed in the original DTI data by the dwidenoise from MRtrix, which implemented dMRI noise level estimation and denoising based on random matrix theory (17, 18). Second, the dwifslpreproc script of MRtrix, which comprises FSL's eddy and top-up tools, was used to correct the eddy current-induced distortion, motion distortion, and susceptibility-induced distortion. Third, DWI bias field correction was performed on data processed in the previous step using the N4 algorithm (19) as provided in ANTs. Fourth, the processed DTI data and the preprocessed T1 image were registered by ANTs (https://github.com/ANTsX) software, and the alignment matrix was obtained. Meanwhile, the alignment matrix was applied into the DTI data. Fifth, applying fiber constrained spherical deconvolution algorithm, orientation distributions were calculated by estimating multitissue response function (20). The whole-brain tractgrams were produced by five-tissue-type segmented T1 image and anatomically constrained tractography. Finally, a 164 × 164 connectivity matrix was produced by mapping the tracks into the 164 nodes. Each contribution to the connectivity was decided by the inverse of the two node volumes, which applied link-based analysis to explore individual connections between any node pair within a network. The procedure is shown in Figure 2.

### Connectivity and Small Network Analysis

Gretna 2.0 was applied for graph theory analysis. In this study, the correlation coefficient matrix is processed into an undirected binary matrix by the sparsity threshold method. The topological organization changes in the whole brain functional network are described by analyzing small-world metrics and network efficiency. The main small-world parameters included the clustering coefficient (Cp) and characteristic path length (Lp), which reflected the mean clustering coefficient and characteristic path length of 100 random networks. The Cp is defined as the average clustering coefficient over all nodes, meanwhile the Lp is defined as the average of all shortest path lengths between all over anode pairs. The network efficiency included global efficiency (Eg), and the mean is the mean local efficiency over all nodes in the network (21). Eg is defined as the inverse of the harmonic mean of the shortest path lengths of each pair of nodes, reflecting the capacity for communications throughout the entire network, while the local efficiency is the mean local efficiency over all nodes in the network. Small-world brain network and network efficiency index between the two groups were compared with two sample *t*-test. The network-based statistic (NBS) was used to identify the group of connections. A total of 5,000 permutations were generated to estimate *p*-values (edge *p* < 0.001, component *p* < 0.05). Meanwhile, a correlation analysis was implemented between the three attentional effects and network efficiency.

## Results

### Demographic and Clinical Information

Socio-demographic characteristics and ANT scores of all recruited subjects are shown in Table 1. Table 1 also shows that the age of patients with SWD distributed between 24 and 33 years old, which can eliminate aging impact on changes in tolerance to shift work. There were also no significant differences in gender and educational level between the two groups.

**Table 1:** The demographic information and ANT between patients with SWD and healthy controls.

**Item**	**SWD** ***N =* 33**	**HC** ***N =* 29**	**χ^2^/*t*/*z***	***p***
Gender (male/female)	2/31	3/26	0.38 (χ^2^)	0.54
Age range (min, max)/years	24, 33	24, 33	NA	NA
Age [M(IQR)]/years	28 (4)	27 (3.5)	−1.66 (*z*)	0.10
Educational level [M(IQR)]/years	16 (3)	16 (3)	−0.56 (*z*)	0.58
Alerting effect [mean(SD)]/ms	39.79 (3.94)	50.34 (3.36)	−2.01 (*t*)	0.049
Orienting effect [M(IQR)]/ ms	44 (23.5)	44 (25)	−0.36 (*z*)	0.72
Executive conflict effect [M(IQR)]/ms	125 (43)	108 (14.5)	−2.44 (*z*)	0.02
Overall mean RT [M(IQR)]/ms	616 (89)	598 (65)	−1.35 (*z*)	0.18
Accuracy [M(IQR)]/%	97 (2.5)	98 (1)	−1.76 (*z*)	0.08

*HC, healthy control; IQR, interquartile range; M, median; RT, reaction time; SD, standard deviation; SWD, shift work disorder*.

The ANT effects between the two groups were conducted *via* two sample t-test analysis or non-parameter test. Compared with healthy controls, patients with SWD showed lower alerting effects and higher executive effects, which suggested declines in alerting and executive functions. No significant differences were observed on orienting effect, overall mean RT, and accuracy between the two groups.

### Group Differences in Connectivity Matrix

**Figure 3:**
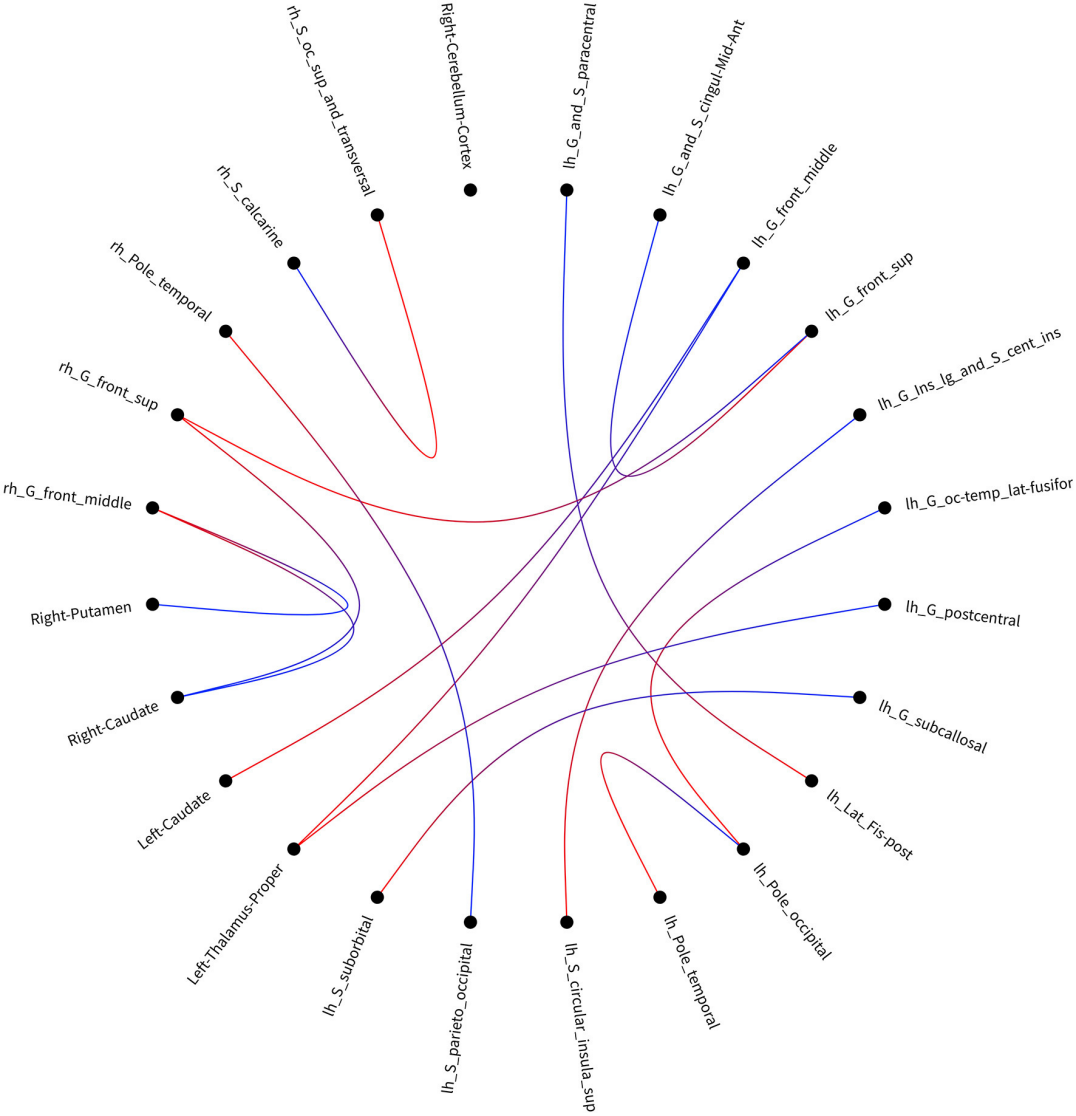
Visualization of the disrupted connectivity matrix found in the group comparison when using link-based analysis. lh, left hemisphere; rh, right hemisphere.

After accumulating 164 × 164 connectivity matrix, we found 15 significant differences in individual graph components between patients with SWD and healthy controls. The results were corrected by NBS (edge *p* < 0.001, component *p* < 0.05). Significant components for patients with SWD showed lower fiber numbers than healthy controls concerned connections between the left proper thalamus and left middle frontal gyrus, between the left proper thalamus and left postcentral gyrus, between the right temporal pole and left parieto-occipital, between the left temporal pole and left occipital pole, between the left caudate and left middle frontal gyrus, between the right middle frontal gyrus and right putamen, between the right superior frontal gyrus and left superior frontal gyrus, between the left superior frontal gyrus and left anterior middle cingulate, between the left occipital pole and left occipital temporal lateral fusiform, between the right middle frontal gyrus and right caudate, between the right superior frontal gyrus and right caudate, between the left circular superior insula and left long insular, between the left suborbital and left subcallosal, between the left lat fissure post and left paracentral, and between the right occipital superior and transversalis and right calcarine (Table 2; Figure 3). No connectivity matrix was found for higher fiber numbers on patients with SWD in contrast with healthy controls.

**Table 2:** Significant components in connectivity matrix between the two groups.

**Components (HC > SWD)**	***t***	***p***
Left proper thalamus–left middle frontal gyrus	3.51	0.03
Left proper thalamus–left postcentral gyrus	3.61	0.03
Right temporal pole–left parieto-occipital	3.65	0.03
Left temporal pole–left occipital pole	4.08	0.01
Left caudate–left middle frontal gyrus	3.62	0.03
Right middle frontal gyrus–right putamen	3.33	0.03
Right superior frontal gyrus–left superior frontal gyrus	3.63	0.03
Left superior frontal gyrus–left anterior middle cingulate	3.5	0.03
Left occipital pole–left occipital temporal lateral fusiform	3.88	0.02
Right middle frontal gyrus–right caudate	3.74	0.02
Right superior frontal gyrus–right caudate	3.84	0.02
Left circular superior insula–left long insular	3.59	0.03
Left suborbital–left subcallosal	3.9	0.02
Left lat fissure post–left paracentral	3.41	0.04
Right occipital superior and transversalis–right calcarine	3.41	0.04

*HC, healthy control; SWD, shift work disorder; –, represents the link between the two brain regions*.

*The results were corrected by NBS (edge p < 0.001, component p < 0.05)*.

### Group Differences in Small-World Parameters and Network Efficiency

**Figure 4:**
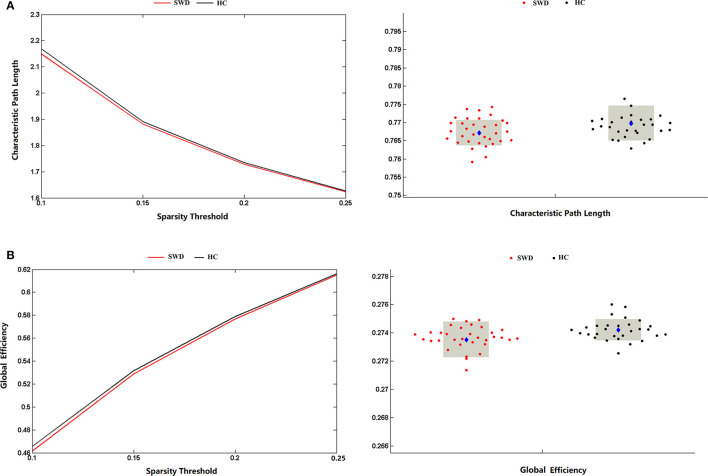
Group comparison of global network topological properties (Lp and Eg) between the patients with SWD and healthy controls. **(A)** The Lp values of the patients with SWD had a significant reduction in contrast with HCs on certain sparsity thresholds (0.1 ≤ Sp ≤ 0.25). **(B)** The result revealed that the patients with SWD exhibited notably lower Eg than the HCs on certain sparsity thresholds (0.1 ≤ Sp ≤ 0.25). Eg, global efficiency; HC, healthy control; Lp, characteristic path length; SWD, shift work disorder.

The matrices were constructed with a wide range of sparsity (0.05–0.5) in all enrolled subjects. The small-world parameters were calculated and further compared between the patients with SWD and healthy controls. The Lp values of the patients with SWD had a significant reduction in contrast with healthy controls (0.1 ≤ Sp ≤ 0.25), which is shown in Figure 4A. After accumulating the global network efficiency, the result revealed that the patients with SWD exhibited notably lower Eg than the healthy controls on certain sparsity thresholds (0.1 ≤ Sp ≤ 0.25), which is shown in Figure 4B. However, no significant differences between the two groups were revealed in regard to the local efficiency.

### Correlation Analysis Between ANT Effects and Network Efficiency

**Figure 5:**
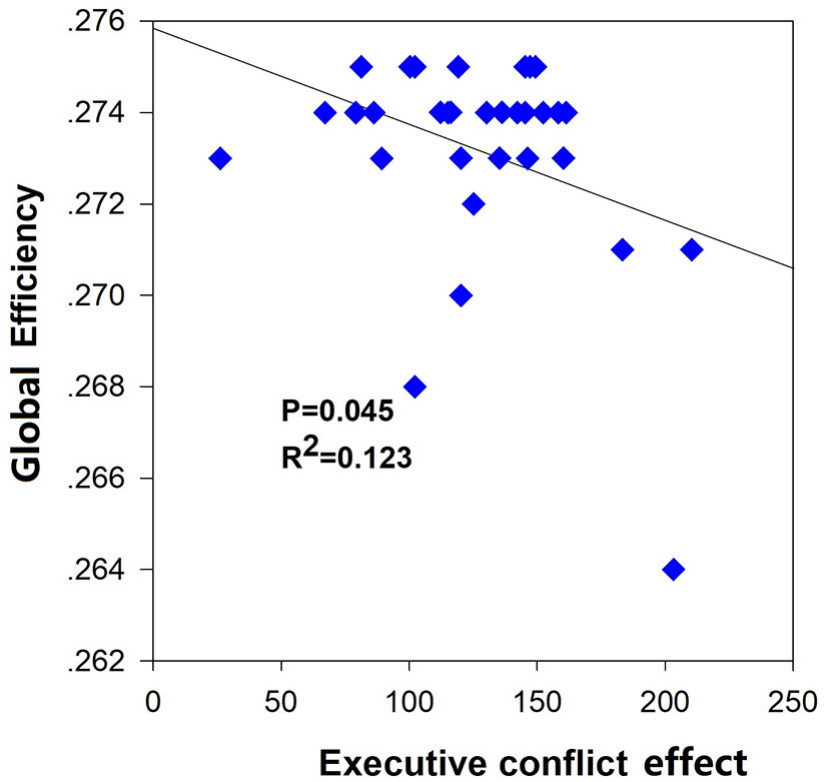
Significant negative correlation between global efficiency and executive conflict effect.

The correlation analysis was conducted between the three attentional effects and the global network efficiency. A significant negative correlation (*R*^2^ = 0.123, *p* = 0.045) was found between the executive effect and global network efficiency (Figure 5).

## Discussion

To our knowledge, this is the first study exploring WM structural connectivity and associations with ANT in patients with SWD. Our results revealed lower alerting effects and higher executive effects for patients with SWD. Using link-based analysis, significant group differences were found for 15 links. Meanwhile, the graph theoretical analysis showed that the patients with SWD displayed declines on the Eg and Lp in contrast with the healthy controls. Moreover, the Eg value was negatively correlated with the executive effect. This study primarily demonstrated the disrupted WM structural brain networks underlying the abnormal characteristic of ANT in SWD.

In our study, we first conducted the comparisons between patients with SWD and healthy controls on efficiency of attentional networks. The lower alerting effects and higher executive effects were found on patients with SWD, which suggested declines in alerting and executive functions. Our results were in line with previous studies. One study on healthcare works showed impaired alertness and performance for night shifts due to circadian misalignment (22). Another study on shift work nurses showed that nurses after working night shifts performed worse concentration and more fatigue compared with after working day shifts (23). Furthermore, it was reported that healthy subjects with mental fatigue impairs pre-attentive processing (24). Hence, we speculated that the fatigue might explain the reasons for declines in alerting and executive functions.

The results from the link-based analysis showed 15 altered connectivity matrices (lower fiber numbers) in patients with SWD compared with healthy controls. The brain regions of disrupted structural connectivity were mainly involved in the frontal gyrus, putamen, and caudate. The frontal gyrus is known to be responsible for emotion processing, working memory, attention, and executive functions (25). One working memory task-related fMRI study on insomnia patients revealed deactivations in the frontal regions (26). In addition, the resting-state fMRI study on insomnia patients showed decreased amplitude of low-frequency fluctuation values in the right middle frontal gyrus and the left orbitofrontal cortex (27). The putamen and caudate belong to the corpus striatum, which forms multisynaptic loops with cortical regions and is associated with motor, memory, and learning. One study on morphological changes in subcortical structures showed that the volume loss of the putamen was associated with impaired cognitive function in patients with chronic insomnia (28). Another study on patients with depression showed disrupted structural connections between the right orbitofrontal cortex and the right putamen, caudate, and inferior temporal gyrus (14). It is possible that the disrupted structural connectivity between frontal gyrus and subcortical nuclei underlies cognitive impairments in patients with SWD. Moreover, we also found lower structural connectivity between the left proper thalamus and left middle frontal gyrus, between the left proper thalamus and left postcentral gyrus. The thalamus is recognized to be involved in maintaining alertness and vigilant attention, which is also a key hub of the cortical attention network (29). The study on sleep deprivation revealed increased thalamic activation after total sleep deprivation, which suggested that increased thalamic activation may compensate for the attention in order to complete the task (30). Notably, the more positive connectivity between the thalamus and cortical regions in patients with insomnia was found in comparison with the healthy controls, which also supported the hyperarousal hypothesis (31). In conclusion, these explorative findings may explain the structural connectivity mechanisms underlying the reduced alertness and executive function in patients with SWD.

The graph theoretical method of structural networks have indicated the shortest paths between brain regions and high clustering of connections, which balanced between the global integration and local specialization. In line with previous studies on structural and functional networks, our results also showed decline on the Eg and Lp between the two groups, which represented the disrupted small-world organization of the structural brain networks. Global network efficiency mainly reflected the capacity for network-wide communication and was regarded as the basis of integrative processing for cognitive functions. Patients with AD (32), primary insomnia (13), and attention deficit hyperactivity disorder (ADHD) (33, 34) all exhibited a decreased global efficiency of the whole WM networks compared with healthy subjects. Nevertheless, the study on sleep deprivation showed the enhanced small-world property, which suggested a possible compensatory effect on the human brain (35). Moreover, the global network efficiency was negatively correlated with the executive effect, which meant the poorer executive function with the lower global efficiency of WM networks. One study on hypertension patients (36) exhibited the executive function impairment underlying a decreased global efficiency of the WM networks. Another study on healthy subjects (12) also showed a significant negative correlation between global efficiency of WM brain network and the executive effect. Hence, we speculated that the declined Eg and Lp may reflect the impaired WM structural network underlying the deficit of attention.

However, there were still some limitations. First, we applied the Destrieux atlas to divide the human brain. However, recent studies have shown that higher-spatial-resolution networks of up to 10,240 parcels (37, 38) could supply an increased sensitivity to local properties. Further studies with higher-spatial-resolution networks may help provide more results on local properties. Second, the enrolled patients in our study only included two male subjects, which failed to analyze the difference in WM structural networks in gender.

## Conclusion

To the best of our knowledge, this is the first study to explore the topological organization of WM structural network connectivity in SWD. Our findings provide new insights into the fundamental architecture of interregional structural connectivity underlying attention deficits in SWD, which may be a potential biomarker for SWD.

## Data Availability Statement

The original contributions presented in the study are included in the article/supplementary material, further inquiries can be directed to the corresponding author/s.

## Ethics Statement

The studies involving human participants were reviewed and approved by Beijing Anding Hospital of Ethics Committee. The patients/participants provided their written informed consent to participate in this study.

## Author Contributions

HJ provided his expertise in shift work disorder, managed the data collection, and contributed to the writing of the manuscript. KL and YN conceived the idea and methodology for the study, designed the study, and contributed to the writing of the manuscript. YN and MF managed data analyses and wrote the manuscript. YZ, SF, ZF, and XL was contributed to conducting the research and the graph display. All authors contributed to the article and approved the submitted version.

## Funding

This study was supported by Beijing Hospitals Authority Youth Program (Grant No. QML20201901), National Natural Science Foundation (Grant No. 81904120 and 82004437), Beijing Natural Science Foundation (Grant No. 7204277), Beijing Hospitals Authority Clinical Medicine Development of Special Funding (Grant No. ZYLX202129), Beijing Hospitals Authority's Ascent Plan (Grant No. DFL20191901), and Talents Training Fund of Beijing (Grant No. 2018000021469G292).

## Conflict of Interest

The authors declare that the research was conducted in the absence of any commercial or financial relationships that could be construed as a potential conflict of interest.

## Publisher's Note

All claims expressed in this article are solely those of the authors and do not necessarily represent those of their affiliated organizations, or those of the publisher, the editors and the reviewers. Any product that may be evaluated in this article, or claim that may be made by its manufacturer, is not guaranteed or endorsed by the publisher.
